# Regulation of anti-phage defense mechanisms by using cinnamaldehyde as a quorum sensing inhibitor

**DOI:** 10.3389/fmicb.2024.1416628

**Published:** 2024-06-26

**Authors:** Antonio Barrio-Pujante, Inés Bleriot, Lucía Blasco, Laura Fernández-Garcia, Olga Pacios, Concha Ortiz-Cartagena, Felipe Fernández Cuenca, Jesús Oteo-Iglesias, María Tomás

**Affiliations:** ^1^Grupo de Microbiología Traslacional y Multidisciplinar (MicroTM)-Servicio de Microbiología Instituto de Investigación Biomédica A Coruña (INIBIC), Hospital A Coruña (CHUAC), Universidad de A Coruña (UDC), A Coruña, Spain; ^2^Study Group on Mechanisms of Action and Resistance to Antimicrobials (GEMARA) the Behalf of the Spanish Society of Infectious Diseases and Clinical Microbiology (SEIMC), Madrid, Spain; ^3^Unidad Clínica de Enfermedades Infecciosas y Microbiología Clínica, Hospital Universitario Virgen Macarena, Instituto de Biomedicina de Sevilla (Hospital Universitario Virgen Macarena/CSIC/Universidad de Sevilla), Sevilla, Spain; ^4^CIBER de Enfermedades Infecciosas (CIBERINFEC), Instituto de Salud Carlos III, Madrid, Spain; ^5^MEPRAM, Proyecto de Medicina de Precisión Contra las Resistencias Antimicrobianas, Madrid, Spain; ^6^Laboratorio de Referencia e Investigación de Resistencias a Antibióticos e Infecciones Sanitarias, Centro Nacional de Microbiología, Instituto de Salud Carlos III, Madrid, Spain

**Keywords:** cinnamaldehyde, phage resistance, *Klebsiella pneumoniae*, proteome, quorum sensing, anti-phage defense mechanisms

## Abstract

**Background:**

Multidrug-resistant bacteria and the shortage of new antibiotics constitute a serious health problem. This problem has led to increased interest in the use of bacteriophages, which have great potential as antimicrobial agents but also carry the risk of inducing resistance. The objective of the present study was to minimize the development of phage resistance in *Klebsiella pneumoniae* strains by inhibiting quorum sensing (QS) and thus demonstrate the role of QS in regulating defense mechanisms.

**Results:**

Cinnamaldehyde (CAD) was added to *K. pneumoniae* cultures to inhibit QS and thus demonstrate the role of the signaling system in regulating the anti-phage defense mechanism. The QS inhibitory activity of CAD in *K. pneumoniae* was confirmed by a reduction in the quantitative expression of the *lsrB* gene (AI-2 pathway) and by proteomic analysis. The infection assays showed that the phage was able to infect a previously resistant *K. pneumoniae* strain in the cultures to which CAD was added. The results were confirmed using proteomic analysis. Thus, anti-phage defense-related proteins from different systems, such as cyclic oligonucleotide-based bacterial anti-phage signaling systems (CBASS), restriction–modification (R–M) systems, clustered regularly interspaced short palindromic repeat-Cas (CRISPR-Cas) system, and bacteriophage control infection (BCI), were present in the cultures with phage but not in the cultures with phage and CAD. When the QS and anti-phage defense systems were inhibited by the combined treatment, proteins related to phage infection and proliferation, such as the tail fiber protein, the cell division protein DamX, and the outer membrane channel protein TolC, were detected.

**Conclusion:**

Inhibition of QS reduces phage resistance in *K. pneumoniae*, resulting in the infection of a previously resistant strain by phage, with a significant increase in phage proliferation and a significant reduction in bacterial growth. QS inhibitors could be considered for therapeutic application by including them in phage cocktails or in phage-antibiotic combinations to enhance synergistic effects and reduce the emergence of antimicrobial resistance.

## Graphical abstract

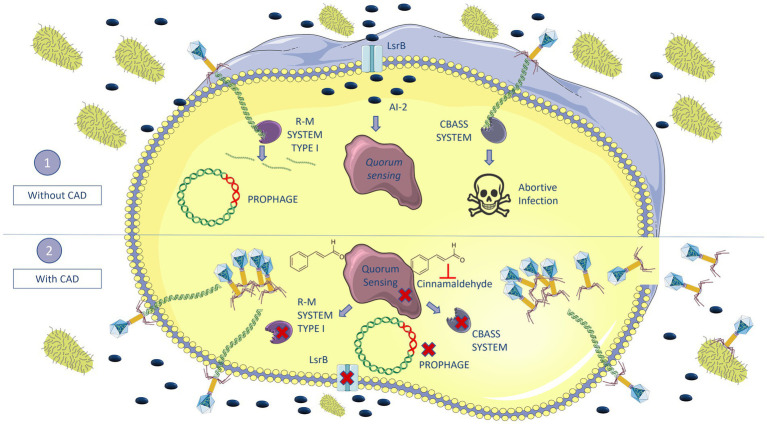

## Introduction

*Klebsiella pneumoniae* is a Gram-negative enterobacteria (Wang et al., 2020), included by the World Health Organization (WHO) in the list of ESKAPE pathogens (*Enterococcus faecium*, *Staphylococcus aureus*, *K. pneumoniae*, *Acinetobacter baumannii*, *Pseudomonas aeruginosa*, and *Enterobacter*). The pathogens pose a serious threat to public health, as these multidrug-resistant (MDR) bacteria can be life-threatening in both hospitalized patients and immunocompromised individuals. Moreover, many of these pathogens can persist stably in biofilms formed on catheters, ventilators, as well as other medical devices (Mancuso et al., 2021).

In this context of clinical urgency, the use of phage therapy has recently re-emerged in the West as one of the main options for treating MDR bacteria (Nikolich and Filippov, 2020). Phage therapy has several advantages over the use of antibiotic therapy. Phages, i.e., viruses that are obligate intracellular parasites of bacteria (Salmond and Fineran, 2015), are highly specific, infecting individual bacterial species or subgroups of species, and are therefore considered narrow-spectrum antimicrobials (Gordillo Altamirano and Barr, 2019; Nikolich and Filippov, 2020). They do not act on the patient’s normal microbiota, they are highly effective against MDR pathogens, and they can be used in conjunction with antibiotics to produce synergetic effects and/or restore the sensitivity of pathogens to antibiotics (Kortright et al., 2019; Gordillo Altamirano et al., 2021). Another advantage of phages is that they can replicate inside the target cell at the site of infection and they are easy to isolate. Moreover, as the most abundant biological entities on the planet, they may represent an inexpensive means of obtaining new antimicrobials (Gordillo Altamirano and Barr, 2019; Nikolich and Filippov, 2020). However, the most important problem regarding phages is the rapid acquisition of resistance in bacteria, as both phages and bacteria are involved in a constant “arms race” to develop resistance and protective mechanisms (Nikolich and Filippov, 2020; Ambroa et al., 2021). In addition, phage therapy also has certain requirements, as the phages must be strictly lytic, have no lysogeny genes such as integrases and recombinases, must have good antibacterial activity to target the pathogen, and bacterial remains and endotoxins must be eliminated (Young and Gill, 2015).

The main mechanisms of phage resistance in bacteria include the following: (i) use of outer membrane vesicles (OMVs) as a decoy for phages, which will inject their DNA into the vesicle (Azam and Tanji, 2019); (ii) blocking phage adsorption to prevent binding to the specific membrane receptor, i.e., by modifying the receptors through mutations (Labrie et al., 2010), or masking them by capsule production or by biofilm formation (Flemming et al., 2023); (iii) blocking nucleic acid injection, i.e., superinfection exclusion (Sies) system (Azam and Tanji, 2019); (iv) degradation of viral nucleic acid—if the phages manage to enter the bacteria, the bacteria may activate defense mechanisms such as exogenous DNA cutting, such as restriction–modification (R–M) (Rusinov et al., 2018), or clustered regularly interspaced short palindromic repeat (CRISPR-Cas) systems (Barrangou et al., 2007); (v) inhibition of phage DNA replication, i.e., by the bacteriophage exclusion (BREX) system (Azam and Tanji, 2019; Rousset et al., 2023); (vi) interference with phage assembly, i.e., phage-inducible chromosomal island (PICI); and (vii) abortive infection (Abi) systems, whereby bacteria being infected by phage cause their own death, thus protecting the rest of the bacterial population and preventing spread of the infection, i.e., toxin/antitoxin (TA) systems and the cyclic oligonucleotide-based antiphage signaling (CBASS) system (Azam and Tanji, 2019; Ambroa et al., 2021).

Many bacteria regulate the expression of defense mechanisms against phages by quorum sensing (QS) (Høyland-Kroghsbo et al., 2017; Rémy et al., 2018). QS is defined as a cellular communication process based on the production, secretion, and detection of extracellular signaling molecules called autoinducers (AIs), which accumulate in the local environment in a cell density-dependent manner (Xavier and Bassler, 2003). QS enables bacteria to regulate several processes such as fluorescence, virulence, biofilm formation, antibiotic resistance, bacterial competition factors, and phage resistance mechanisms such as prophages, CBASS system, and CRISPR-Cas immunity (Galloway et al., 2011; Broniewski et al., 2021; Lazar et al., 2021; Pacheco et al., 2021; Severin et al., 2023).

The QS of *K. pneumoniae* mainly relies on the action of autoinducer-2 (AI-2), a furanosyl borate diester molecule encoded by LuxS synthase, for interspecies communication. Moreover, this species can detect other AIs in the medium, such as exogenous *N*-acyl homoserine lactone (AHL), known as AI-1 (Lazar et al., 2021; Pacheco et al., 2021).

In this study, the role of QS in controlling anti-phage defense mechanisms was tested in *K. pneumoniae* by using the QS inhibitor cinnamaldehyde (CAD). CAD has previously been shown to interfere with the AI-2-based QS *Vibrio* spp. and *Burkholderia* spp. by reducing the DNA binding activity of the response regulator LuxR (Brackman et al., 2008, 2009, 2011). It also possesses antimicrobial activity and can inhibit both microbial growth and toxin production (Doyle and Stephens, 2019); it has been reported to exert antiviral activity against influenza A/PR/8 virus by inhibiting viral protein synthesis at the post-transcriptional level thereby reducing the growth of the virus (Hayashi et al., 2007). CAD can cause allergic reactions when included in perfumes and cosmetics, and the dermatological no-observed-adverse-effect level has been established as 0.5% (Shreaz et al., 2016). In addition, when inhaled in e-cigarettes, CAD has been shown to be toxic to lung cells by inhibiting cell growth and differentiation, increasing cell death and DNA strand breaks (Behar et al., 2016; Zhu et al., 2017).

Finally, CAD has been recognized by both the Flavor and Extract Manufacturers Association (FEMA) and the U.S. Food and Drug Administration (FDA) as a safe compound (Friedman, 2017).

In this study, the role of the QS in the anti-phage defense was determined by the use of CAD to sensitize a phage-resistant *K. pneumoniae* clinical isolate.

## Materials and methods

### Bacterial and phage strains

Two lytic phages that infect *K. pneumoniae* were used in the study: the vB_KpnM_VAC36 phage (VAC36) (Family *Myoviridae* and Genus *Marfavirus*) and the vB_KpnM_VAC66 phage (VAC66) (Family *Myoviridae* and Genus *Slopekvirus*) (Bleriot et al., 2023). The genomes of both phages are available via the GenBank BioProject with accession number PRJNA739095: VAC36 (GenBank SAMN20298872) and VAC66 (GenBank SAMN22059211).

Three clinical isolates of *K. pneumoniae* with different sensitivity to the phages were used: K3318, a phage-resistant clinical isolate (GenBank SAMEA3649518); K3573, a clinical isolate sensitive to phage VAC36 (GenBank SAMEA3649559); and ST974-OXA48, a clinical isolate sensitive to phage VAC66 (GenBank WRWT00000000). All clinical isolates of *K. pneumoniae* were obtained from the Virgen Macarena University Hospital (Seville, Spain) and the National Center for Microbiology (Carlos III Health Institute, Spain).

### Propagation and collection of phages

Phages were propagated by the double-layer agar method (Da Silva et al., 2021). An overnight inoculum of each of the different strains of *K. pneumoniae* (phage host) was diluted 1/100 in Luria–Bertani (LB) broth and grown until the absorbance at 600 nm (optical density, OD_600_) reached 0.5. Aliquots of 50 μL of phage were then added to 200 μL of the corresponding *K. pneumoniae* host and mixed with 4 mL of soft agar (0.5% NaCl, 1% tryptone, and 0.4% agar) in TA plates (0.5% NaCl, 1% tryptone, and 1.5% agar) and incubated at 37°C for 24 h. The TA plates were washed with 3 mL of SM buffer (0.1 M NaCl, 10 mM MgSO_4_, and 20 mM Tris–HCl) and placed on a shaker at room temperature for 3 h. All of the liquid was then recovered in 15 mL tubes, to which 1% chloroform was added, and the mixture was held for 20 min. Finally, the suspension was centrifuged for 15 min at 3,400 × *g* and filtered through 0.45 nm filters. The supernatant with the phages was collected and stored at 4°C.

### Cinnamaldehyde minimal inhibitory concentration assay

**Figure 1:**
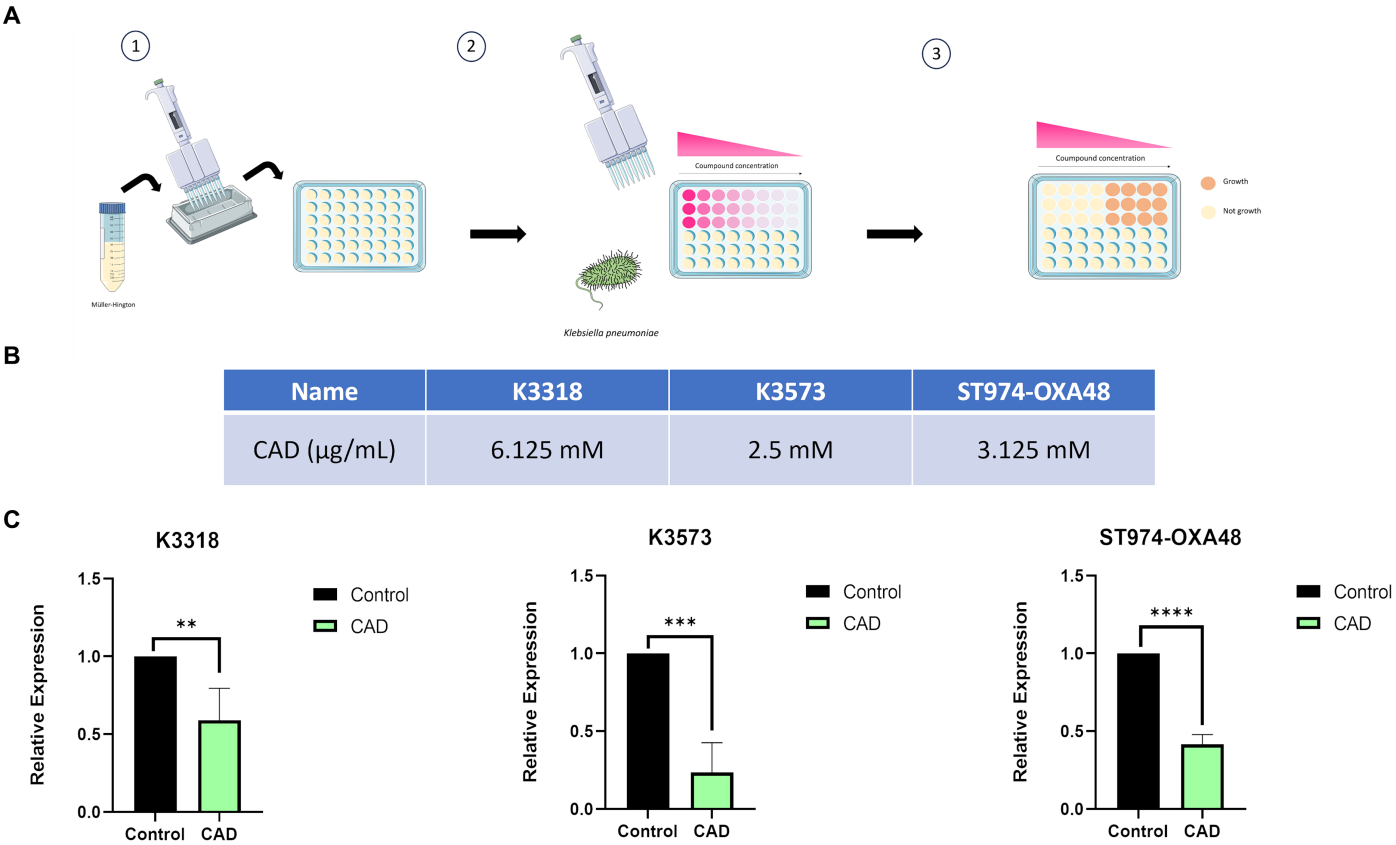
Illustration of the microdilution broth assay used to determine the MICs of Cinnamaldehyde (CAD) and to quantify *lsrB* gene expression. **(A)** Illustration of the microdilution broth assay process for different strains of *K. pneumoniae* in the presence of CAD. **(B)** MICs of CAD for different strains of *K. pneumoniae*. **(C)** Quantification of *lsrB* gene expression in the presence of 1 mM CAD relative to the control. The asterisks refer to statistical significance, the more asterisks, the more significant the difference between the groups compared. *p < 0,0332; **p < 0,0021; ***p < 0,0002; ****p < 0,0001.

The CAD (3-phenylprop-2-enal; Sigma-Aldrich) minimum inhibitory concentrations (MICs) for *K. pneumoniae* clinical strains K3318, K3573, and ST974-OXA48 were established by microdilution broth assay (Wikler and Clinical, 2006; Pacios et al., 2021). Briefly, nine serial double dilutions of CAD were prepared in Muller–Hinton broth (MHB) in 96-well microtiter plates. Finally, each well was then inoculated with the corresponding strain of *K. pneumoniae* to a final concentration of 5 × 10^5^ CFU/mL, diluted from an overnight culture. A row of MHB inoculated with *K. pneumoniae* was included as a positive control and a row including only MHB was included as a negative control. The plates were incubated for 24 h at 37°C, and finally, the MICs were determined as the concentration of CAD in the first well where no bacterial growth was observed (Pacios et al., 2021). All experiments were performed in triplicate (Figure 1A).

### Quantitative expression of the *lsrB* gene

**Figure 2:**
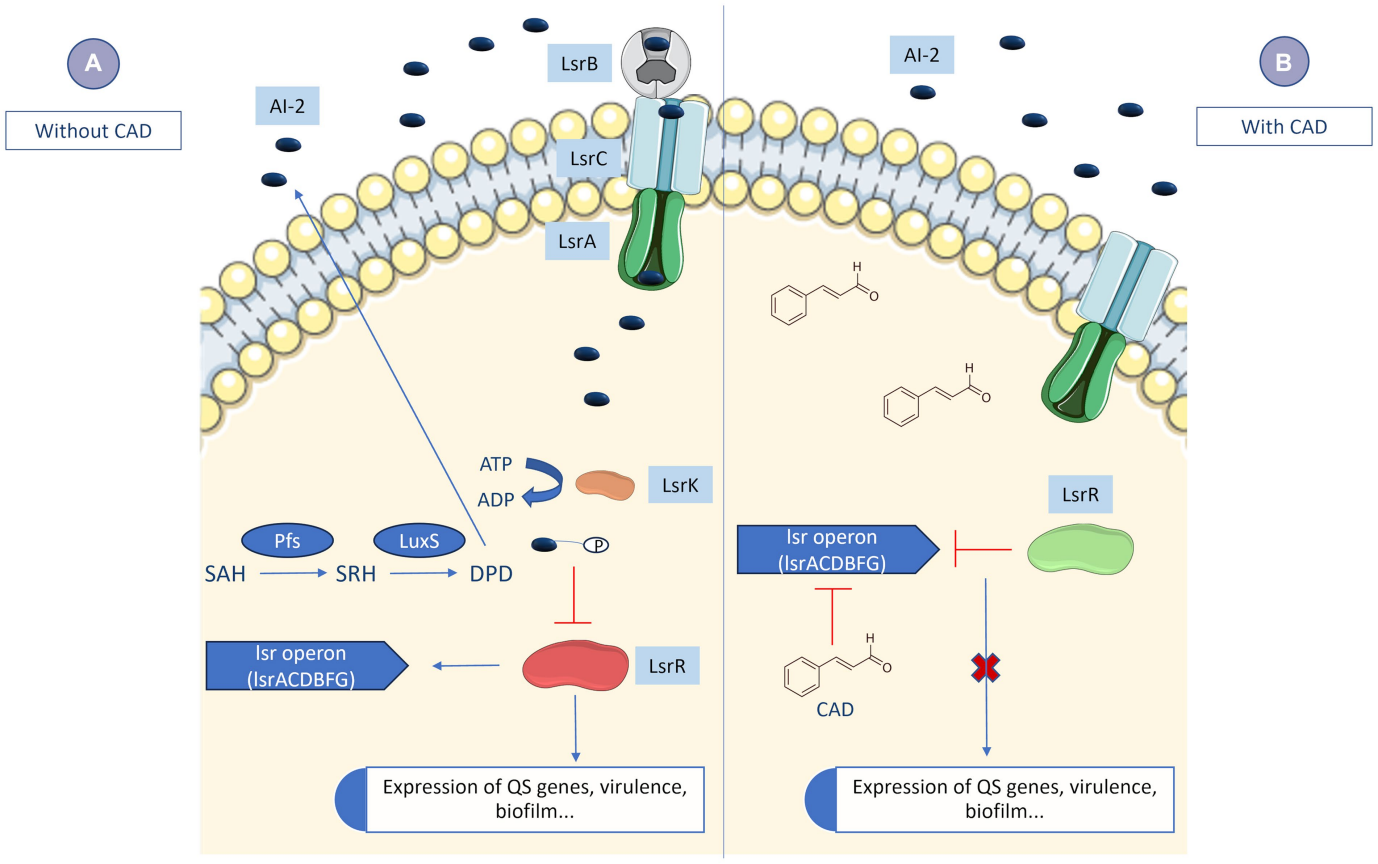
QS inhibition by the action of CAD. **(A)** Under normal conditions, the bacteria synthesize AI-2 in the extracellular environment. AI-2 from the extracellular environment is internalized via LsrB into the bacteria. Subsequently, the AI-2 will be phosphorylated by LsrK. The phosphorylated AI-2 molecule represses LsrR (repressor of the *lsrACDBFG operon*) so that much more AI-2 begins to be transported into the bacteria. Transport of AI-2 into the cell, together with the repression of LsrR, leads to the transcription of numerous genes related to bacterial virulence, biofilm, QS, etc. **(B)** When cinnamaldehyde (CAD) is inside the cell, expression of the *lsrB* gene is inhibited, preventing extracellular AI-2 from being internalized. Thus, LsrR itself continues to inhibit the *lsrACDBFG operon,* and transcription of the aforementioned genes does not occur, so the QS cascade is inhibited.

Expression of the *lsrB* gene, which is responsible for transporting AI-2 into the cell (Figure 2), was quantified in the presence of a subinhibitory concentration of CAD (1 mM) in the three clinical isolates of *K. pneumoniae.* For this purpose, 200 μL of an overnight culture of the corresponding *K. pneumoniae* clinical isolate was inoculated in 20 mL flasks of LB broth containing 1 mM CAD (two biological samples of each strain). The flasks were then incubated at 37°C with shaking at 180 rpm, until an OD_600_ of 0.3 was reached. Duplicate aliquots of 1 mL were taken from each flask for subsequent RNA extraction with the High Pure RNA Isolation Kit (Roche). The extraction was conducted following the manufacturer’s instructions. The RNA extracted from each sample was measured in a Nanodrop spectrophotometer (NanoDrop Technologies) and adjusted to 50 ng/μL with nuclease-free water for use in qRT-PCR (LightCycler^®^ 480). Specific primers for the *lsrB* gene and its corresponding UPL probe and specific primers for the *rho* gene, included as a housekeeping gene (Gomes et al., 2018), with the corresponding UPL probe (Table 1), were used in the assay (conducted with the LightCycler^®^ 480 Control Kit from Roche). Student’s *t*-test (GraphPad Prism 9.0.0) was used to determine any statistically significant differences (*p*-value < 0.05) in gene expression.

**Table 1:** Primers and probes used for *rho* and *lsrB* genes in the qRT-PCRs.

Primer name	Sequences	Probes	References
***lsrB* gene (AI-2 transport)**			
Kp_lsrB_Fow	CAGTATGAGGCGAAGGGCAA	74	This study
Kp_lsrB_Rev	TCGTAGTTGTTGATGTTCTCTTTGC	74	This study
***rho* gene (Housekeeping gene)**			
Kp_rho_Fow	CGACGGCGTACTGGAGATAC	17	This study
Kp_rho_Rev	TTGGCTGGGGGATACGTAGA	17	This study

### Phage infection assays

Phage infection curves were constructed for strains K3318, ST974-OXA48, and K3573, and phages VAC36 and VAC66. Briefly, an overnight culture of the *K. pneumoniae* strain to be tested was diluted 1/100 in LB broth. Five different conditions were prepared with the bacterial culture at an initial OD_600_ of 0.3: a growth control; *K. pneumoniae* culture with 1 mM CAD; *K. pneumoniae* strain with the corresponding phage at a multiplicity of infection (MOI) 1; *K. pneumoniae* strain in combination with the phage at MOI 1 and 1 mM CAD; and a negative control with LB broth. A final volume of 200 μL was added to each corresponding well of 96-well plates.

The plates were incubated at 37°C with shaking at 559 cycles per minute (CPM) for 24 h in a BioTek Epoch 2.0 (Agilent). The contents of three wells for each condition were recovered at different time points, and the bacteria and phages were quantified by counting colony-forming units (CFUs/mL) and plaque-forming units (PFUs/mL) at 0 h (T0), 5 h (T5), and 24 h (T24). To quantify the CFUs, 100 μL of culture was removed at the appropriate time and diluted to the correct dilution for counting the cells. Finally, 100 μL of the dilution was plated on LB plates. For the quantification of PFUs, the phages were isolated as described below. At the corresponding time points, 100 μL of culture was removed, 1% chloroform was added, and the mixture was shaken for 20 min. The suspension was then centrifuged at 10,000 × *g* for 5 min and serially diluted to the correct dilution for counting. Finally, the double-layer agar method was used to obtain the PFUs (Da Silva et al., 2021); the plates were incubated for 24 h at 37°C, and the CFUs/mL and PFUs/mL were counted. All experiments were performed in triplicate. Student’s *t*-test was used to determine any statistically significant differences (*p* < 0.05) in the bacteria counts (GraphPad Prism 9.0.0).

### Proteomic analysis

A proteomic study was conducted to determine the inhibition of anti-phage defense mechanisms and QS by using LC–MS and NanoUHPLC-Tims-QTOF analysis.

Strain K3318 (obtained from an overnight inoculum) was inoculated into 50 mL flasks containing LB broth at 1/100 dilution, and the culture was incubated until an OD_600_ of 0.3 (approximately 1 × 10^7^ CFU/mL) was reached. The four previously described treatments were tested in duplicate. The culture strains were allowed to grow for 3 h, before 25 mL of the suspension was removed from each flask, placed in 50 mL tubes, and placed on ice for 10 min. The tubes were centrifuged for 20 min at 4°C and 4,500 rpm, and the pellet obtained after discarding the supernatant was frozen at −80°C. On the following day, the pellet was resuspended in phosphate-buffered saline (PBS) medium and sonicated with an ultrasonic processor (UP200S, Hielscher Ultrasonics) at an amplitude of 80% and a cycle of 0.5 per second for periods of 90 s with the sample on ice. The sonicated pellets were centrifuged for 20 min at 4°C at 4,500 × *g*. The supernatant was recovered and used in the proteomic analysis.

A quantitative analysis of proteins was conducted with 200 ng of supernatant from each sample using the NanoUHPLC-Tims-QTOF in a TimsTof Pro mass spectrophotometer (Bruker), with a nanoESI source (CaptiveSpray), a QTOF-time analyzer and a nanoELUTE chromatograph (Bruker). The samples were prepared by tryptic digestion in solution with reduction-alkylation, followed by ZipTip desalting. The data were obtained in a nanoESI positive ionization mode, Scan PASEF-MSMS mode, and CID fragmentation mode, with an acquisition range of 100–1,700 m/z. The products were separated on a ReproSil C18 column (150 × 0.075 mm, 1.9 μm, and 120 Å) (Bruker) at 50°C with an injection volume of 2 μL. The mobile phases consisted of 0.1% H_2_O/formic acid (A) and 0.1% acetonitrile/formic acid (B). The flow rate was 0.4 μL/min, and the gradient program was 11% B (0–5 min), 16% B (5–10 min), 35% B (10–16 min), 95% B (16–18 min), and 95% B (18–20 min). Finally, different types of software were used for data acquisition: Compass HyStar 5.1 (Bruker), TimsControl (Bruker), DataAnalysis (Bruker), and PEAKS studio (Bioinformatics Solutions).

Student’s *t*-test was used to detect any statistically significant differences (*p*-value < 0.05) in the results (GraphPad Prism 9.0.0).

## Results

### CAD microdilution broth assay

The microdilution broth assay was used to determine the MICs of CAD for clinical isolates of *K. pneumoniae* (Figure 1B). The MICs were 6.125 mM for clinical strain K3318, 2.5 mM for clinical strain K3573, and 3.125 mM for clinical strain ST974-OXA48.

### Quantitative expression of the *lsrB* gene

In order to confirm the role of CAD in inhibiting the *K. pneumoniae* QS, the expression of the AI-2 QS periplasmic transporter *lsrB* gene (Figure 2) was measured (Zhang et al., 2020). Expression of *lsrB* was significantly lower in the presence of CAD than in the absence of CAD (Figure 1C). This difference was observed for the three isolates of *K. pneumoniae* used in the study, thus confirming the inhibitory effect of CAD on QS in *K. pneumoniae.*

### Phage infection assays

**Figure 3:**
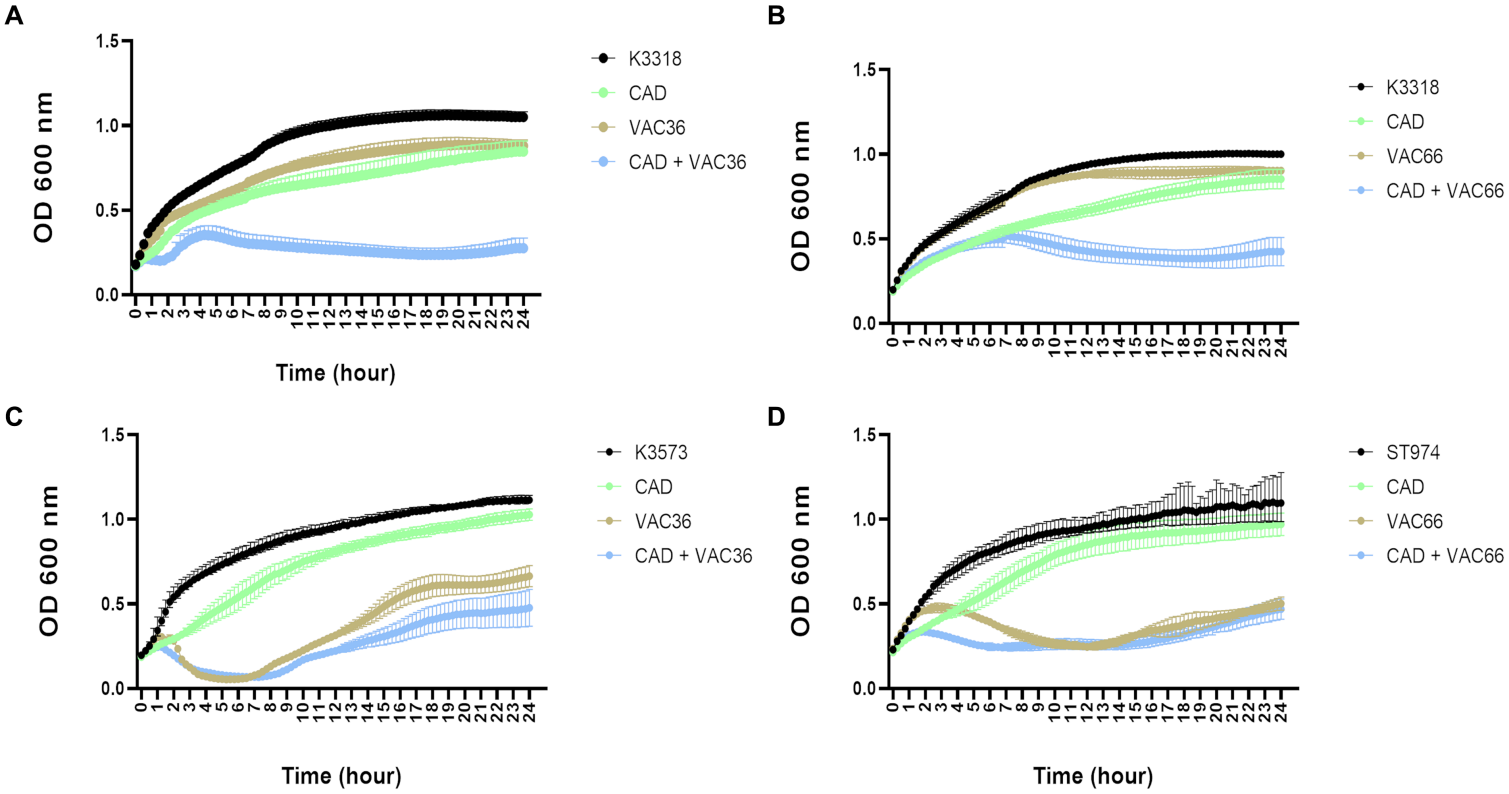
Infection curves for the three clinical isolates of *K. pneumoniae* with phage alone and phage in combination with CAD. **(A)** Strain K3318 infected with VAC36. **(B)** Strain K3318 infected with VAC66. **(C)** Strain K3573 infected with VAC36. **(D)** Strain ST974-OXA48 infected with phage VAC66.

**Figure 4:**
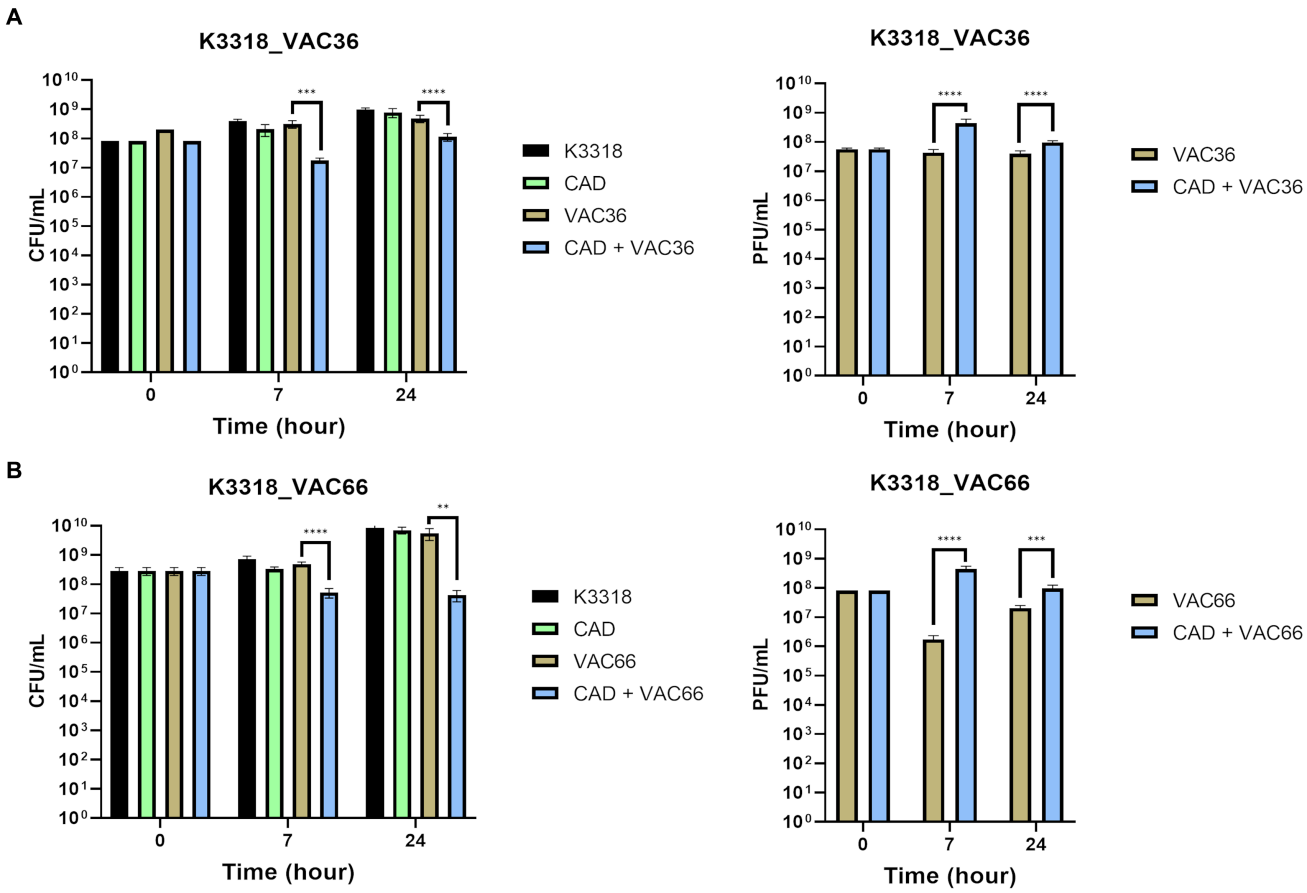
Quantification of bacteria and phages by CFU/mL and PFU/mL counts in phage infection assays. **(A)** CFU/mL and PFU/mL of strain K3318 infected with phage VAC36. **(B)** CFU/mL and PFU/mL of strain K3318 infected with phage VAC66. The asterisks refer to statistical significance, the more asterisks, the more significant the difference between the groups compared. *p < 0,0332; **p < 0,0021; ***p < 0,0002; ****p < 0,0001.

The phage infection assays were performed to test the infectivity of phages in the presence of the QS inhibitor CAD. The corresponding infection curves showed a decrease in the OD_600_ of strain K3318 when the bacteria were infected with the phages in combination with CAD (Figures 3A,B), as this strain was resistant to both phages. The control infection curve showed similar growth. In the sensitive strains, K3573 and ST974-OXA48, no differences were observed when the bacteria were infected with the phage alone or in combination with CAD (Figures 3C,D). A slight reduction in initial growth was observed in the CAD condition in all three strains. To confirm the OD_600_ results, CFUs and PFUs were counted at 0 h, 7 h, and 24 h (Figures 4A,B). The results revealed a significant decrease in the number of CFUs at 7 h and a significant increase in the PFUs for strain K3318 when infected with the combination of CAD and each of the phages. No decrease in CFUs or increase in PFUs was observed when the bacteria were infected with each phage alone. At 24 h, the number of CFUs was higher in the cultures with CAD plus phage but significantly lower than in the control with phage and growth control. At 24 h, although the number of PFUs was lower, it was significantly higher than the number corresponding to each phage alone. No significant differences in CFUs between the control group and the CAD condition were observed. These results are consistent with a productive phage infection after the addition of CAD, suggesting that CAD favors phage infection by inhibiting bacterial defense mechanisms.

Host strains K3573 and ST974-OXA48 were sensitive to phage infection, but there were no significant differences between the treatment with phage alone and the treatment with CAD plus phage (Figures 3C,D). Owing to the lack of any differences, the CFUs and PFUs were not quantified. As the phage was able to infect the culture in the control condition, it seems that no efficient bacterial defense mechanisms were activated in this case, and the CAD did not have any effect.

### Study of the proteins related to anti-phage defense mechanisms and QS

**Figure 5:**
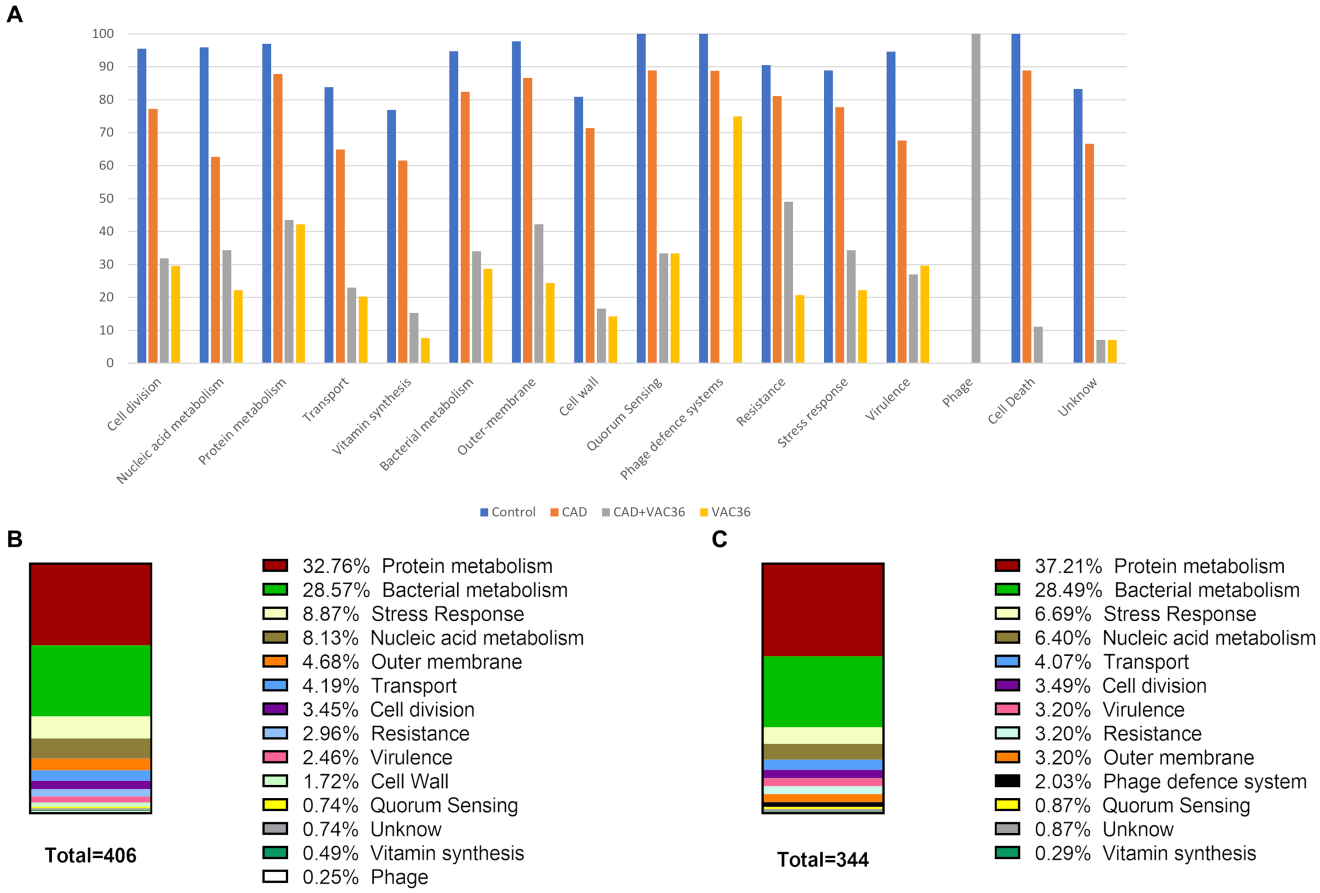
Graphical representation of proteomic analysis. **(A)** Relative abundance of each functional protein group in K3318. **(B)** Abundance of proteins belonging to each functional group in the CAD plus VAC36 treatment. **(C)** Abundance of proteins belonging to each functional group in the VAC36 treatment. Parts of the figure were drawn using diagrams available from Servier Medical Art. Servier Medical Art by Servier is licensed under a Creative Commons Attribution 3.0 Unported License (https://creativecommons.org/licenses/by/3.0/).

A proteomic analysis (NanoUHPLC-Tims-QTOF) was conducted to confirm that the anti-phage defense mechanisms are regulated by QS. The analysis identified a total of 1,222 proteins among all samples, grouped according to function and abundance across all conditions (Supplementary Table S1; Figure 5A). For example, 406 proteins were identified in the CAD plus VAC36 treatment, and 344 proteins were identified in the condition with phage alone (Figures 5B,C).

The analysis showed that the main representative proteins in all conditions were related to bacterial metabolism and protein metabolism. These proteins were less abundant in the treatments, including phage (Supplementary Table S1; Figures 5A–C). Although the relative abundance of proteins related to nucleic acid metabolism was reduced in the treatment with phage, it was slightly higher in the CAD plus VAC36 treatment (Figure 5A). Interestingly, the analysis revealed a higher relative abundance of proteins related to anti-phage defense when CAD was not added to the culture. Proteins related to functions such as cell division, the outer membrane, and cell wall production were also less abundant in the treatment with phage (Figure 5A). The effect of CAD on phage infection was highlighted, as in this condition only phage-related proteins were detected. In addition, the outer membrane proteins, which usually act as phage receptors, were also more abundant in this treatment relative to the treatment with phage alone (Figure 5A).

Proteins related to anti-phage defense mechanisms were present when the culture was only infected with phage and was absent in the treatment, including CAD (Table 2). The defense system proteins identified belong to the CBASS system, such as purine-nucleoside phosphorylase (WFM04862.1), uridine phosphorylase (WGN84707.1), ubiquitin-like protein (WP_289465582.1), and polyubiquitin (WP_223807730.1). They also belonged to the CRISPR-Cas type I-E defense system, such as CRISPR-associated protein Cas7/Cse4/CasC (WAY94155.1), to the R-M system, including restriction endonuclease subunit S (WAD08528.1), and finally to a prophage-related defense system known as bacteriophage control infection (BCI) (UZL08287.1), previously described by our research group (Ambroa et al., 2020). In addition, a tail phage protein (WP_153932364.1) was only present in significant amounts when both phage and CAD were added to the culture. Other proteins, such as the cell division protein DamX (UZL52976.1), which contributes to more efficient infection of the phage, and the outer membrane channel protein TolC (WBL85855.1), a phage receptor, were more abundant in the cultures to which CAD was included than in the corresponding cultures without the compound, confirming active infection. These results confirmed the regulatory activity of the QS in the anti-phage defense mechanisms.

**Table 2:** Proteins associated with quorum sensing (QS), anti-phage defense mechanisms, and phage proliferation, detected in the proteomic analysis.

Quorum related								
Description	Accession no.	−10LgP	Control	CAD	CAD + VAC	VAC	Mechanism	References
(4S)-4-hydroxy-5-phosphonooxypentane-2,3-dione isomerase [*Klebsiella pneumoniae*]	UZL69991.1	1.48E+07	7.07E+06	1.93E+07	0.00E+00	0.00E+00	Quorum Quenching	Marques et al. (2011)
Alpha/beta fold hydrolase [*Klebsiella pneumoniae*]	WCN21377.1	1.39E+07	8.80E+06	3.04E+07	0.00E+00	0.00E+00	Quorum Quenching	Sikdar and Elias (2020)
LuxR C-terminal-related transcriptional regulator, partial	WP_265783091.1	9.21E+06	1.18E+07	0.00E+00	0.00E+00	0.00E+00	Quorum Sensing	Pacheco et al. (2021)
Ribosome-associated translation inhibitor RaiA	UZL23362.1	2.08E+07	4.61E+07	2.22E+05	0.00E+00	0.00E+00	Persistence	Lang et al. (2021)
Anti-phage defense								
Type I-E CRISPR-associated protein Cas7/Cse4/CasC	WAY94155.1	2.02 + 06	1.66E+07	0.00E+00	0.00E+00	0.00E+00	CRISPR-Cas	Barrangou et al. (2007), Makarova et al. (2011)
Bacteriophage Control Infection (BCI)	UZL08287.1	1.87E+07	1.43E+07	3.22E+07	0.00E+00	1.93E+06	Virulence	Ambroa et al. (2020)
Purine-nucleoside phosphorylase	WFM04862.1	2.46E+06	7.40E+07	1.56E+07	0.00E+00	1.88E+07	CBASS System	Rousset et al. (2023)
Uridine phosphorylase	WGN84707.1	1.88E+07	4.51E+07	1.61E+07	0.00E+00	2.42E+06	CBASS System	Rousset et al. (2023)
Uridine phosphorylase	WBY12188.1	1.44E+07	1.57E+05	1.68E+07	0.00E+00	1.44E+06	CBASS System	Rousset et al. (2023)
Restriction endonuclease subunit S	WAD08528.1	6.03E+07	1.53E+06	1.77E+07	0.00E+00	2.32E+07	R-M System	Loenen et al. (2014)
Ubiquitin-like protein, partial	WP_289465582.1	1.32E+07	2.05E+07	4.45E+07	0.00E+00	6.72E+06	CBASS System	Jenson et al. (2023)
Polyubiquitin, partial	WP_223807730.1	2.02E+07	6.70E+06	7.49E+06	0.00E+00	6.72E+06	CBASS System	Jenson et al. (2023)
Phage related								
Tail fiber domain-containing protein	WP_153932364.1	1.73E+07	0.00E+00	0.00E+00	4.65E+07	0.00E+00	Tail phage	Olszak et al. (2017)
Cell division protein DamX	UZL52976.1	2.79E+07	1.86E+07	8.05E+06	3.90E+07	1.54E+06	Cell Division	Wenzel et al. (2024)
Outer membrane channel protein TolC	WBL85855.1	1.90E+07	2.89E+07	5.16E+07	7.23E+06	0.00E+00	Phage receptor	Hasan and Ahn (2022)

Interestingly, the abundance of protein confirmed the inhibitory effect of CAD on QS. Thus, quorum quenching proteins related to QS extinction, such as (4S)-4-hydroxy-5-phosphonooxypentane-2,3-dione isomerase (UZL69991.1) and alpha/beta fold hydrolase (WCN21377.1), were also more abundant in the cultures with CAD than in the control culture. LuxR (WP_265783091.1), a known QS receptor, was only present in the control culture, and the ribosome-associated translation inhibitor RaiA (UZL23362.1), another protein of interest related to bacterial persistence, was less abundant in the presence of CAD.

## Discussion

Phage therapy provides a great opportunity to advance in the fight against MDR bacteria; phages can be used alone and in cocktails, and they can also be used in combination with antibiotics to restore bacterial sensitivity to these agents (Chan et al., 2016; Blasco et al., 2023). Phages have many advantages over conventional antimicrobials: they are cheap and easy to obtain, highly specific and self-replicating, and they do not act on the patient’s normal microbiota (Gordillo Altamirano and Barr, 2019; Kortright et al., 2019; Nikolich and Filippov, 2020; Gordillo Altamirano et al., 2021). However, the main problem with their use is the rapid acquisition of resistance in bacteria, which possess numerous defense mechanisms against phages (Nikolich and Filippov, 2020; Ambroa et al., 2021). Therefore, inhibiting anti-phage defense mechanisms, currently achieved by combining both antibiotic and phage therapies, is an important strategy to improve phage therapy, although the current strategy only works for those bacteria with phage receptors involved in antibiotic resistance (Blasco et al., 2019, 2023; Cesta et al., 2023; Bleriot et al., 2024).

In this context, the present study aimed to determine whether anti-phage resistance mechanisms are regulated by QS in *K. pneumoniae*. Previous studies have shown that QS regulates anti-phage defense mechanisms in bacteria under conditions of high cell density: the bacteria are more exposed to phages as the viruses are more abundant in densely populated environments, as they need bacteria to proliferate (Høyland-Kroghsbo et al., 2013).

In the present study, the relationship between QS and the anti-phage defense mechanism was tested by inhibiting QS with CAD. The QS autoinductor in *K. pneumoniae* is the AI-2 synthesized by an ortholog of LuxS synthase, which is also related to biofilm production (Balestrino et al., 2005). The relationship between anti-phage defense and QS has been demonstrated in *Escherichia coli* in a study in which the AI-2 induced a defensive response against phages by decreasing both the overall host metabolism and the phage receptors (Ding et al., 2021).

Inhibition of QS in clinical isolates of *K. pneumoniae* was confirmed in the present study by the *lsrB* gene, known to encode an AI-2 transporter with a previously demonstrated role in anti-phage defense (Zhang et al., 2020; Ding et al., 2021). The role of CAD in QS inhibition was demonstrated in uropathogenic *E. coli* strains, in a study in which CAD reduced QS and biofilm production (Capper-Parkin et al., 2023). The inhibitory effect of CAD on QS has also been demonstrated in other bacteria such as *Vibrio* spp. and *Burkholderia* spp. (Brackman et al., 2008, 2009, 2011; Capper-Parkin et al., 2023). The inhibitory role of CAD was established in the present study for *K. pneumoniae,* as a 60% decrease in *lsrB* expression levels was observed when CAD was present at subinhibitory concentrations (1 mM) (Figure 1C).

The role of QS in regulating anti-phage defense mechanisms was previously demonstrated by Hoque et al., who observed that QS in *V. cholerae* (via cholera AI-1- and AI-2-like AIs) regulates the production of haemagglutinin protease (HAP), which is responsible for inactivating viral particles and deregulating phage receptors, particularly the LPS O-antigen receptor, thereby preventing phage adhesion (Hoque et al., 2016). Moreover, the high cell density caused by QS in *V. cholerae* cultures has been associated with the transcription of two essential components of the CBASS defense system, the oligonucleotide cyclase and the phospholipase effector (Severin et al., 2023). Numerous studies have confirmed the activation of the CRISPR-Cas defense system at high cell densities. Thus, in *Serratia* spp., an increase in type I-E, I-F, and III CRISPR-Cas systems was shown to be caused by QS regulation (Patterson et al., 2016). In *P. aeruginosa*, QS was demonstrated to be involved in regulating CRISPR-Cas genes and activating three key aspects of the system, namely expression, activity, and adaptation (Høyland-Kroghsbo et al., 2017; Broniewski et al., 2021). In addition, it has been suggested that *P. aeruginosa* regulates cell death by using the Pseudomonas quinolone signal (PQS) to prevent the spread of phage infection (Shah et al., 2021). In *E. coli*, activation of QS was shown to reduce the number of phage λ receptors on the cell surface in order to evade infection (Høyland-Kroghsbo et al., 2013; Ding et al., 2021). Finally, the Brex defense system is also linked to QS through S-adenosyl methionine (SAM), a precursor in the synthesis of AI-2 that may be a necessary cofactor in the system (Xavier and Bassler, 2003; Andriianov et al., 2023). We are not aware of any studies specifically linking *K. pneumoniae* QS to anti-phage defense mechanisms such as those described above.

The role of QS in the expression of anti-phage defense mechanisms was tested by infecting the phage-resistant strain *K. pneumoniae* K3318 with two different phages in combination with 1 mM CAD. The infection curves obtained for two phages showed a significant reduction in the OD_600_ when resistant strain K3318 was infected with phages in combination with CAD (Figures 3A,B), as well as a reduction in the CFU/mL and an increase in the PFU/mL after 7 h and 24 h (Figures 4A,B). As isolate K3318 was initially resistant to phages VAC36 and VAC66, the results of the control infection were similar to those obtained for the growth control. In the sensitive strains (Figures 3C,D), no resistance was observed as both strains were killed by their respective phages, and no effect of phage in combination with CAD was observed as the results of infection in the presence and absence of CAD were similar. In the growth curves for the presence of CAD alone, the growth of both strains was slightly reduced. This effect was also observed for the resistant strain K3318, but the difference was not large enough to be reflected in the CFU counts. Although the antimicrobial activity of CAD has been demonstrated (Doyle and Stephens, 2019), it seems that this effect was negligible at the subinhibitory concentration used in the present study. The antiviral activity of the CAD was also established for the influenza virus but was not observed in the present study (Hayashi et al., 2007), as the CAD did not affect infection of the sensitive strains; in the case of the resistant strain K3318, the presence of CAD favored the infection, and the counts of PFUs increased, thus indicating increased phage replication and inhibition of defense mechanisms.

The results of the proteomic study confirmed those revealed by the infection curve and the relationship between QS and anti-phage defense mechanisms. Moreover, the CAD-mediated QS inhibition was validated by the lower or higher expression of some proteins related to QS in the cultures with CAD than in the control culture (Table 2). Some of these proteins are directly related to quenching of the QS signal and were found to be more abundant in the presence of CAD, e.g., (4S)-4-hydroxy-5-phosphonoxypoxypentane-2,3-dione isomerase, which is directly involved in the degradation of phospho-AI-2, as it converts the molecule (4S)-4-hydroxy-5-phosphonoxypoxypentane-2,3-dione (P-DPD) (a precursor of the AI-2) into 3-hydroxy-5-phosphonoxypoxypentane-2,4-dione (P-HPD), closing the signaling cycle and leading to termination of the expression of the *lsr operon* (Marques et al., 2011). The alpha/beta-fold hydrolase protein shows a preference for a wide variety of AHL substrates and may have lactonase activity (Sikdar and Elias, 2020). The orphan LuxR C-terminal-related transcriptional regulator, another protein related to AI receptors, was not present in cultures with CAD or phage. In *K. pneumoniae*, this protein detects (but does not produce) exogenous AHL from other bacterial species and also regulates virulence factors such as biofilm formation, expression of fimbriae, cell division, and production of QS AIs (Pacheco et al., 2021). Finally, the ribosome-associated translation inhibitor RaiA, a ribosome-associated protein related to the transition to the stationary phase, was less abundant in the cultures to which CAD was added than in the control culture, owing to persistence mechanisms (Lang et al., 2021).

The relationship between QS and phage resistance was also reflected by the abundance of some phage-related proteins present when the CAD was added along with the phage. The relative abundance of proteins related to DNA metabolism was slightly higher when the cultures were infected with CAD plus VAC36 than when they were infected with the phage alone (Figure 5A), probably due to active replication of the phage. In the phage infection, the number of proteins related to cell division, cell wall, or outer membrane was reduced. However, in cultures to which CAD was added along with the phage, the outer membrane proteins were almost two times more abundant than in cultures infected with phage alone. One of the main anti-phage defense mechanisms is receptor masking (Bleriot et al., 2024). The proteins that act as receptors were present in the cultures to which CAD was added, as no defense mechanisms were activated; however, the same proteins were undetectable in the cultures to which only phage was added, as a result of receptor masking. These proteins include some outer membrane proteins (Hantke, 2020), in particular the outer membrane channel protein TolC (Table 2), which has been shown to act as a phage receptor in *E. coli* (phage TLS) and *Salmonella* spp. (phages ST27, ST29, and ST35) (Hasan and Ahn, 2022). The cell division protein DamX, an important component of the episome, has been reported to play a supportive role during phage replication by increasing replication efficiency (Wenzel et al., 2024). Although this protein is more abundant in the CAD condition due to phage replication, it is also present in other conditions because it is involved in cell division (Wenzel et al., 2024) (Table 2). The absence of resistance and the active infection in the presence of CAD were finally confirmed by the presence of a tail phage protein, which was not present in the cultures without CAD (Table 2), i.e., during the absence of infection as observed in the infection curves (Figures 3A,B). The presence of this protein in the synergistic interaction between CAD and phage shows that a productive infection was taking place in this treatment and new phage progeny was being produced (Olszak et al., 2017). The proteins related to anti-phage defense mechanisms were only detected when the infection was established in the absence of CAD, which suggests that CAD is involved in inhibiting the anti-phage defense mechanism. Many of these proteins belong to the CBASS defense system, an Abi system composed of an oligonucleotide cyclase that is responsible for activating the effector upon sensing the infection and a cyclic oligonucleotide-sensitive effector that kills the infected cell (Jenson et al., 2023; Rousset et al., 2023; Severin et al., 2023). The CBASS proteins were present in the cultures infected with phage alone, but not in the cultures in which QS was inhibited by the addition of CAD. The CBASS proteins identified were purine-nucleoside phosphorylase, uridine phosphorylase, ubiquitin-like protein, and polyubiquitin. The two first proteins act as effector proteins in the CBASS, as both are purine nucleoside phosphorylases (PNP), forming part of the cyclic oligonucleotide-sensitive effector (Rousset et al., 2023). Ubiquitin and polyubiquitin are involved in coding *cap2*, a gene whose catalytic activity is essential for the correct functioning of the CBASS system (Jenson et al., 2023). The restriction endonuclease subunit S protein, which is related to the R-M defense system, was also absent in cultures to which CAD was added along with phage (Mruk and Kobayashi, 2014). The R-M system provides a type of innate immunity that protects prokaryote cells from the insertion of foreign DNA, including a restriction endonuclease (REase), which recognizes and cuts short DNA sequences, and a methyltransferase (Mtase), which methylates host DNA so that it cannot be cut by REase (Birkholz et al., 2022). The restriction endonuclease subunit S belongs to the R–M type I system, which is encoded by three genes: *hsdR,* which encodes the restriction subunit (R); *hsdM*, which encodes the modification subunit (M); and *hsdS*, which encodes the recognition subunit (S for specificity) (Loenen et al., 2014). A CRISPR-associated protein Cas7/Cse4/CasC, a bacterial defense-related protein, was detected in the control cultures and belongs to the CRISPR-Cas type I–E system (Makarova et al., 2011). The absence of this protein in the phage-only condition, in which the anti-phage defense mechanisms are active, may be explained by the mode of action of the CRISPR-Cas system, which needs to carry a specific spacer for the phage, and if it does not (because there has been no previous contact between the phage and bacteria), the system would not be activated (Makarova et al., 2011). The BCI protein, a carbon storage family protein present in prophages and involved in controlling infection by other phages, was also detected. Expression of BCI has been shown to favor the activation of the QS (Ambroa et al., 2020). This protein was totally inhibited by the combination of CAD and phage and present in all other conditions, possibly because its expression is regulated by QS, and when inhibited, it cannot provide protection against phage infection.

Finally, in the cultures to which phage was added along with CAD, multiple resistance mechanisms, such as efflux pumps, were activated (Figure 5A; Supplementary Table S1). This finding can probably be attributed to the presence of CAD. In *P. aeruginosa*, exposure to subinhibitory concentrations of CAD resulted in the expression of efflux pump encoding operons (Tetard et al., 2019).

The present study demonstrated that bacterial defense mechanisms are directly linked to and controlled by QS in *K. pneumoniae*. Inhibition of QS reduces phage resistance, and inhibitory compounds could be included in phage cocktails or in phage-antibiotic combinations, potentially further enhancing the synergy and reducing the emergence of resistance. This would partly resolve the drawback of using phages by themselves.

The study findings indicate that QS inhibition appears to be a promising strategy for evading bacterial defense. However, a limited number of phages and bacterial strains were tested in the present study, and an examination of the effect of CAD on a larger number of phages and phage-resistant strains is recommendable. QS inhibition may be the missing piece of the puzzle that will enable the great potential of phages to be fulfilled, thus providing a possible solution to the ever-increasing problem of MDR bacteria and the lack of antibiotics.

## Data availability statement

The original contributions presented in the study are included in the article/Supplementary material, further inquiries can be directed to the corresponding author.

## Author contributions

AB-P: Investigation, Methodology, Writing – original draft. IB: Supervision, Validation, Writing – review & editing. LB: Supervision, Validation, Writing – review & editing, Investigation. LF-G: Investigation, Writing – review & editing, Visualization. OP: Writing – review & editing, Data curation, Validation. CO-C: Data curation, Validation, Writing – review & editing, Conceptualization, Visualization. FC: Visualization, Writing – review & editing, Resources. JO-I: Visualization, Writing – review & editing, Investigation, Supervision, Validation. MT: Investigation, Supervision, Validation, Visualization, Writing – review & editing, Funding acquisition, Methodology.
